# Sunburn Prevalence Among US High School Students, Youth Risk Behavior Survey, United States, 2023

**DOI:** 10.5888/pcd23.260069

**Published:** 2026-05-28

**Authors:** Dawn M. Holman, Ryan Saelee, Sandra J. Kiplagat, Anne K. Julian

**Affiliations:** 1 Centers for Disease Control and Prevention, Division of Cancer Prevention and Control, Atlanta, Georgia; 2 Centers for Disease Control and Prevention, Division of Adolescent and School Health, Atlanta, Georgia

## Abstract

Using 2023 national Youth Risk Behavior Survey data, we estimated the prevalence of any sunburn and frequent sunburn (≥5 times in the past year) among US high school students and examined associations with demographic and behavioral characteristics. Overall, 54.7% of students reported any sunburn, and 22.9% of students with any sunburn reported frequent sunburn. After adjustment for sex, race and ethnicity, and grade, any sunburn prevalence differed by sex, race and ethnicity, body mass index, several substance use and activity measures, diet, and social media use; factors associated with frequent sunburn partially overlapped but also differed. Persistently high adolescent sunburn prevalence supports continued youth skin cancer prevention efforts.

**Summary**

**What is already known on this topic?**

Sunburn prevalence among US high school students has remained persistently high.

**What is added by this report?**

In 2023, 54.7% of US high school students reported sunburn in the past year. In adjusted analyses, sunburn prevalence differed significantly by sex, race and ethnicity, body mass index, tobacco use, alcohol use, marijuana use, fruit and vegetable consumption, physical activity, muscle-strengthening exercise, and social media use. In unadjusted analyses, prevalence also differed by mental health status.

**What are the implications for public health practice?**

Persistently high sunburn prevalence among adolescents supports continued skin cancer prevention efforts in schools and in health care and community settings.

## Objective

Sunburn from solar ultraviolet radiation or indoor tanning devices is a well-established risk factor for skin cancer (1). Research findings indicate that sunburn during adolescence increases a person’s risk for melanoma (2), basal cell carcinoma (3), and squamous cell carcinoma (2) in adulthood. Approximately 6.1 million people in the US are treated for skin cancer each year, at a cost of nearly $9 billion (4). Preventing sunburn during adolescence is therefore a public health priority (1). Healthy People 2030 includes an objective to “reduce the proportion of students in grades 9 through 12 who report sunburn” (C-10), with a target prevalence of 52.2% (5). To monitor progress toward this objective, a question about the number of sunburns experienced in the past 12 months was included in the 2023 national Youth Risk Behavior Survey (YRBS) (6,7). Previous analyses of national YRBS data found high sunburn prevalence among US high school students (8). We estimated the current prevalence of any sunburn and frequent sunburn and examined associations with selected demographic, behavioral, and health characteristics among US high school students.

## Methods

We analyzed cross-sectional, self-reported data from the 2023 national YRBS, a nationally representative school-based survey of US students in grades 9 through 12 (9). Students were asked, “During the past 12 months, how many times have you had a sunburn? (Count the number of times even a small part of your skin turned red or hurt for 12 hours or more after being outside in the sun or after using a sunlamp or other indoor tanning device).” Response options ranged from 0 times to 5 or more times. We defined any sunburn as 1 or more sunburns and frequent sunburn as 5 or more sunburns among students reporting any sunburn. Covariates were sex, grade, race and ethnicity, body mass index , tobacco use, alcohol use, marijuana use, trying to lose weight, fruit and vegetable consumption, physical activity, muscle-strengthening exercise, social media use, and mental health.

Because of small cell sizes, the frequent sunburn analysis combined non-Hispanic American Indian or Alaska Native, Asian, Black, Native Hawaiian or Other Pacific Islander, Hispanic, and multiracial students into 1 category. We used R version 4.5.0 with the R survey package (version 4.4-2) (10) to account for the complex sampling design and weighting. We estimated weighted prevalence and 95% CIs and used pairwise *t* tests for unadjusted group comparisons. For each outcome, we fit separate quasi-Poisson models with log links for each characteristic, adjusting for sex, race and ethnicity, and grade (selected a priori as standard demographic confounders in YRBS analyses); 2-sided *P* < .05 was considered significant. Centers for Disease Control and Prevention (CDC) reviewed this activity, deemed it not research, and determined it was conducted consistent with applicable federal law and CDC policy (45 CFR part 46.102(l)(2), 21 CFR part 56; 42 USC §241(d); 5 USC §552a; 44 USC §3501 et seq.).

## Results

The 2023 national YRBS sample included 20,103 students. The overall response rate was 35.4% (school response rate, 49.8%; student response rate, 71.0%). Among 10,788 students who answered the sunburn item, 54.7% reported at least 1 sunburn in the past 12 months (Table 1). 

**Table 1:** Prevalence of Any Sunburn in the Past 12 Months Among US High School Students, by Demographic, Behavioral, and Health Characteristics, National Youth Risk Behavior Survey, 2023^a^

Characteristic	Students reporting any sunburn, no.	Unadjusted % (95% CI)	Adjusted PR (95% CI)
**Total**	5,868	54.7 (49.6–59.8)	—
**Sex**			
Male	2,753	52.0 (45.9–58.0)^b^	1 [Reference]
Female	3,096	57.9 (52.8–62.9)	1.14 (1.08–1.20)
**Grade**			
9	1,498	53.2 (48.5–57.9)	1 [Reference]
10	1,557	56.3 (50.6–61.8)	1.02 (0.95–1.08)
11	1,407	54.3 (47.4–61.0)	1.00 (0.93–1.08)
12	1,377	55.9 (49.4–62.3)	0.99 (0.92–1.06)
**Race and ethnicity**			
NH American Indian or Alaska Native	391	42.3 (29.7–56.1)^c^	2.89 (2.06–4.06)
NH Asian	132	31.1 (24.6–38.5)^d^	2.19 (1.69–2.83)
NH Black or African American	179	14.3 (11.9–17.1)^e^	1 [Reference]
Hispanic or Latino	1,009	41.4 (36.3–46.7)^f^	2.86 (2.28–3.60)
NH Native Hawaiian or other Pacific Islander	21	45.7 (24.1–69.0)^g^	3.14 (1.97–5.00)
NH White	3,523	78.7 (75.9–81.4)^h^	5.51 (4.55–6.67)
NH Multiracial	561	45.6 (37.9–53.5)	3.19 (2.55–4.00)
**Body mass index**			
Underweight (<5th percentile)	177	50.4 (41.3–59.5)^i^	0.86 (0.74–1.01)
Healthy weight (5th–<85th percentile)	3,576	58.4 (53.5–63.2)^j^	1 [Reference]
Overweight (85th–<95th percentile)	815	52.7 (45.8–59.4)	0.98 (0.91–1.05)
Obese (≥95th percentile)	883	49.1 (43.4–54.9)	0.92 (0.87–0.98)
Tobacco use in past 30 days			
Yes	1,309	62.2 (56.7–67.3)^k^	1.08 (1.02–1.15)
No	4,249	53.6 (48.0–59.2)	1 [Reference]
**Alcohol use in past 30 days**			
Binge drinking^l^	659	77.0 (71.9–81.5)^m^	1.27 (1.17–1.37)
Non–binge drinking only	623	59.5 (53.4–65.3)^n^	1.10 (1.03–1.18)
None	4,227	51.5 (45.9–57.0)	1 [Reference]
**Marijuana use in past 30 days**			
Yes	1,175	59.0 (53.8–63.9)^o^	1.07 (1.02–1.12)
No	4,643	54.0 (48.5–59.5)	1 [Reference]
**Was trying to lose weight**			
Yes	2,724	53.7 (48.5–59.0)	0.97 (0.93–1.01)
No	3,094	55.7 (50.2–61.1)	1 [Reference]
**Ate ≥1 fruit (or fruit juice) and ≥1 vegetable per day**			
Yes	1,457	60.2 (55.3–64.9)^p^	1.10 (1.05–1.15)
No	4,343	53.5 (47.9–59.0)	1 [Reference]
**Physically active ≥60 min/day**			
Yes	1,678	60.6 (54.9–66.0)^q^	1.07 (1.02–1.13)
No	4,137	53.0 (47.7–58.1)	1 [Reference]
**Muscle strengthening exercise ≥3 days per week**			
Yes	3,098	59.4 (53.7–64.8)^r^	1.15 (1.10–1.21)
No	2,721	50.6 (45.5–55.6)	1 [Reference]
**Social media use**			
At least several times per day	4,800	56.5 (51.6–61.2)^s^	1.13 (1.05–1.21)
About once per day or less	1,019	50.0 (42.3–57.6)	1 [Reference]
**Poor mental health most of the time or always in past 30 days**			
Yes	1,944	58.0 (53.0–62.8)^t^	0.99 (0.94–1.05)
No	3,860	53.8 (48.3–59.3)	1 [Reference]

Abbreviations: —, not applicable; PR, prevalence ratio (adjusted for sex, race and ethnicity, and grade); NH, non-Hispanic.

a N = 20,103 respondents. The number of students answering each question varied. Data were missing because the question did not appear on a student’s questionnaire, the student did not answer the question, or the response was out of range or logically inconsistent. Percentages in each category were calculated from nonmissing data. After adjustment for sex, race and ethnicity, and grade, a total of 10,788 students answered the sunburn item. Numbers in the “Students reporting any sunburn” column reflect the count of students in each subgroup who reported any sunburn (numerator); denominators vary by characteristic and are not shown.

b Male students significantly differed from female students (*P* = .008), based on *t* test with Taylor series linearization.

c NH American Indian or Alaska Native students significantly differed from NH Black or African American (*P* < .001) and NH White (*P* < .001) students, based on *t* test with Taylor series linearization.

d NH Asian students significantly differed from NH Black or African American (*P* < .001), Hispanic or Latino (*P* = .014), NH White (*P* < .001), and NH Multiracial (*P* < .001) students, based on *t* test with Taylor series linearization.

e NH Black or African American students significantly differed from Hispanic or Latino (*P* < .001), NH Native Hawaiian or Other Pacific Islander (*P* = .008), NH White (*P* < .001), and NH Multiracial (*P* < .001) students, based on *t* test with Taylor series linearization.

f Hispanic or Latino students significantly differed from NH White (*P* < .001) students, based on *t* test with Taylor series linearization.

g NH Native Hawaiian or Other Pacific Islander students significantly differed from NH White (*P* = .005) students, based on *t* test with Taylor series linearization.

h NH White students significantly differed from NH Multiracial (*P* < .001) students, based on *t* test with Taylor series linearization.

i Students who were underweight significantly differed from students with healthy weight (*P* = .04), based on *t* test with Taylor series linearization.

j Students with healthy weight significantly differed from students with overweight (*P* = .012) and obesity (*P* < .001), based on *t* test with Taylor series linearization.

k Students who used tobacco in the past 30 days significantly differed from students who did not (*P* = .001), based on *t* test with Taylor series linearization. Tobacco use included smoking cigarettes or cigars, using smokeless tobacco products, and using electronic vapor products.

l Had 4 or more drinks of alcohol in a row (if they were female) or 5 or more drinks of alcohol in a row (if they were male) within a couple of hours on ≥1 day during the 30 days before the survey.

m Students who reported binge drinking in the past 30 days significantly differed from students who did non–binge drinking (*P* < .001) and those who did not drink (*P* < .001), based on *t* test with Taylor series linearization.

n Students who reported non–binge drinking in the past 30 days significantly differed from students who did not drink (*P* = .003), based on *t* test with Taylor series linearization.

o Students who reported using marijuana in the past 30 days significantly differed from students who did not (*P* = .03), based on *t* test with Taylor series linearization.

p Students who ate ≥1 fruit (or fruit juice) and ≥1 vegetable per day during the past 7 days significantly differed from students who did not (*P* < .001), based on *t* test with Taylor series linearization.

q Students who were physically active ≥60 min per day during the past 7 days significantly differed from students who did not (*P* < .001), based on *t* test with Taylor series linearization.

r Students who did muscle strengthening exercise ≥3 days per week during the past 7 days significantly differed from students who did not (*P* < .001), based on *t* test with Taylor series linearization.

s Students who used social media at least several times per day significantly differed from students who used it about once per day or less (*P* = .010), based on *t* test with Taylor series linearization.

t Students who reported poor mental health most of the time or always in the past 30 days significantly differed from students who did not (*P* = .013), based on *t* test with Taylor series linearization.

In unadjusted analyses (*P* < .05), any sunburn differed by sex, race and ethnicity, body mass index, tobacco use, alcohol use, marijuana use, fruit and vegetable consumption, physical activity, muscle-strengthening exercise, social media use, and mental health. After adjustment for sex, race and ethnicity, and grade, any sunburn prevalence was higher among female students; students reporting tobacco use, alcohol use, or marijuana use; students reporting consuming at least 1 fruit (or fruit juice) and at least 1 vegetable per day; students reporting physical activity of 60 or more minutes per day; students reporting muscle-strengthening exercise at least 3 days per week; and students using social media at least several times per day. Any sunburn prevalence was lower among students with obesity than among students with healthy weight. In adjusted analyses, non-Hispanic Black or African American students served as the reference group; all other racial and ethnic groups had significantly higher adjusted sunburn prevalence (Table 1).

Among students reporting any sunburn, 22.9% reported frequent sunburn (≥5 sunburns) (Table 2). After adjustment, frequent sunburn was more prevalent among female students, non-Hispanic White students, students reporting binge drinking, physically active students, and students reporting muscle-strengthening exercise at least 3 days per week, and less prevalent among 12th-grade students than 9th-grade students. 

**Table 2:** Prevalence of Frequent Sunburn (≥5 Sunburns) in the Past 12 Months Among US High School Students Reporting Any Sunburn, by Demographic, Behavioral, and Health Characteristics, National Youth Risk Behavior Survey, 2023^a^

Characteristic	Students reporting frequent sunburn, no.	Unadjusted % (95% CI)	Adjusted PR (95% CI)
**Total**	1,389	22.9 (19.2–27.0)	—
**Sex**			
Male	558	19.8 (16.2–23.9)^b^	1 [Reference]
Female	830	26.0 (21.6–31.0)	1.36 (1.17–1.58)
**Grade**			
9	364	23.5 (18.3–29.6)	1 [Reference]
10	387	25.5 (20.4–31.4)	1.03 (0.80–1.33)
11	332	22.5 (17.5–28.4)	0.92 (0.70–1.23)
12	290	19.1 (15.6–23.1)^c^	0.73 (0.57–0.93)
**Race and ethnicity** d			
Other racial and ethnic groups combined	328	9.9 (8.0–12.2)	1 [Reference]
NH White	1,052	29.1 (25.1–33.5)	3.03 (2.47–3.73)
**Body mass index**			
Underweight (<5th percentile)	33	17.1 (9.6–28.7)	0.81 (0.49–1.34)
Healthy weight (5th–<85th percentile)	867	23.2 (19.2–27.9)	1 [Reference]
Overweight (85th–<95th percentile)	179	20.0 (16.2–24.5)	0.90 (0.76–1.06)
Obese (≥95th percentile)	200	21.8 (17.8–26.4)	1.01 (0.86–1.19)
**Tobacco use in past 30 days**			
Yes	354	26.8 (22.6–31.5)	1.25 (0.97–1.63)
No	956	21.8 (17.3–27.1)	1 [Reference]
**Alcohol use in past 30 days**			
Binge drinking^e^	214	30.4 (24.5–37.1)^f^	1.49 (1.14–1.97)
Non–binge drinking only	149	23.1 (19.6–26.9)	1.13 (0.90–1.42)
None	926	21.4 (16.7–27.0)	1 [Reference]
**Marijuana use in past 30 days**			
Yes	288	25.3 (20.3–30.9)	1.21 (0.94–1.56)
No	1,091	22.4 (18.1–27.4)	1 [Reference]
**Was trying to lose weight**			
Yes	649	23.0 (19.3–27.2)	1.00 (0.87–1.16)
No	725	22.7 (18.6–27.5)	1 [Reference]
**Ate ≥1 fruit (or fruit juice) and ≥1 vegetable per day**			
Yes	383	24.2 (19.6–29.5)	1.10 (0.96–1.25)
No	982	22.1 (18.5–26.2)	1 [Reference]
**Physically active ≥60 min per day**			
Yes	493	28.0 (23.2–33.3)^g^	1.37 (1.17–1.61)
No	875	20.5 (16.9–24.7)	1 [Reference]
**Muscle strengthening exercise ≥3 days per week**			
Yes	765	25.3 (20.8–30.4)^h^	1.34 (1.13–1.59)
No	611	20.1 (16.5–24.2)	1 [Reference]
**Social media use**			
At least several times per day	1,168	23.2 (19.8–27.0)	1.24 (1.00–1.55)
About once per day or less	194	19.0 (13.2–26.7)	1 [Reference]
**Poor mental health most of the time or always in past 30 days**			
Yes	515	24.8 (20.5–29.6)	1.07 (0.85–1.33)
No	853	21.7 (17.4–26.7)	1 [Reference]

Abbreviations: —, not applicable; PR, prevalence ratio (adjusted for sex, race and ethnicity, and grade); NH, non-Hispanic.

a Among students reporting any sunburn in the past 12 months (N = 5,868).

b Male students significantly differed from female students (*P* < .001) based on *t* test with Taylor series linearization.

c Students in 12th grade significantly differed from 10th-grade (*P* = .013) and 11th-grade (*P* = .04) students, based on *t* test with Taylor series linearization.

d Race and ethnicity categories other than NH White were combined because of small cell sizes; this category includes NH American Indian or Alaska Native, NH Asian, NH Black or African American, Hispanic or Latino, NH Native Hawaiian or Other Pacific Islander, and NH multiracial students. Students of other racial and ethnic groups significantly differed from NH White (*P* < .001) students, based on *t* test with Taylor series linearization.

e Had 4 or more drinks of alcohol in a row (if they were female) or 5 or more drinks of alcohol in a row (if they were male) within a couple of hours on ≥1 day during the 30 days before the survey.

f Students who reported binge drinking in the past 30 days significantly differed from students who did not drink (*P* = .02), based on *t* test with Taylor series linearization.

g Students who were physically active ≥60 min per day significantly differed from students who were not (*P* < .001), based on *t* test with Taylor series linearization.

h Students who did muscle strengthening exercise ≥3 days per week during the past 7 days significantly differed from students who did not (*P* = .02), based on *t* test with Taylor series linearization.

## Discussion

More than half of US high school students reported sunburn in 2023, and nearly one-quarter of students with any sunburn reported 5 or more sunburns. The prevalence of any sunburn (54.7%) exceeded the Healthy People 2030 target of 52.2% (objective C-10), suggesting that additional prevention efforts may be needed to meet this national goal.

The higher prevalence among female students than male students contrasts with adult patterns, in which adjusted sunburn prevalence does not typically differ meaningfully by sex (11). Differences by race and ethnicity may reflect, in part, variation in sun sensitivity and skin tone and are consistent with the higher skin cancer incidence observed among non-Hispanic White populations (12).

Consistent with adult sunburn data (11), sunburn data for high school students showed association with several behavioral factors. These patterns may reflect clustering of risk behaviors and greater outdoor exposure among more physically active students. The lower sunburn prevalence observed among students with obesity may reflect less time spent outdoors, and the positive association with fruit and vegetable consumption may reflect clustering with other outdoor health–oriented behaviors. Both findings should be interpreted cautiously given the potential for residual confounding. We also observed an association between frequent social media use and any sunburn. We are not aware of prior national YRBS sunburn analyses that examined social media use; future studies should assess whether this association reflects appearance norms, tanning attitudes, outdoor behaviors, or exposure to prevention messages.

Strengths of this study include a nationally representative sample of US high school students and examination of social media use, a relatively unexplored factor in the sun-safety literature. Limitations include self-reported data, which are subject to recall and social desirability bias; limited adjustment for potential confounders; inability to distinguish incidental sun exposure from intentional outdoor tanning or indoor tanning device use; the school-based design, which may limit generalizability to adolescents not attending high school; and incomplete data for the sunburn item. In addition, sunburn data were missing for 9,315 respondents (46.3% of the total sample). Because the pattern of missingness is unknown, some nonresponse bias is possible. 

Persistently high sunburn prevalence among US high school students supports continued youth skin cancer prevention efforts. Interventions that integrate sun protection with broader adolescent health promotion may be useful, particularly for groups with higher observed sunburn prevalence. These findings can inform future research and intervention efforts.
